# Development and psychometric validation of the PERQOLATEUR questionnaire: an individualized self-report measure of the impact of chronic illness and treatment in clinical practice

**DOI:** 10.1186/s41687-026-01102-4

**Published:** 2026-06-09

**Authors:** Jean-Benoit Hardouin, Djuly Pierre-Paul, Anne Le Rhun, Philippe Tessier, Marianne Bourdon, Leila Moret

**Affiliations:** 1 INSERM UMR 1236-SPHERE Methods in Patients Centered Outcomes and Health Research, University of Nantes, University of Tours, Nantes, France; 2 Service de Santé Publique et Environnementale (SPPE), Centre Hospitalier Universitaire de Nantes, Nantes, France; 3 Institut de Cancérologie de l’Ouest, Nantes, France

**Keywords:** Quality of life, Individualized self-questionnaire, Clinical practice, Chronic disease, Disease-agnostic questionnaire

## Abstract

**Background:**

Quality of life is a key construct in healthcare, particularly in the concept of health-related quality of life (HRQoL). Existing questionnaires are widely used in research but remain underused in clinical practice, partly due to their length and lack of personalization. No self-administered HRQoL instrument currently combines rapid completion with results that are immediately interpretable by clinicians. This study aimed to develop and validate a concise, self-administered, French-language personalized HRQoL questionnaire.

**Methods:**

A multidisciplinary working group, including patients, designed and validated the PerQolateur questionnaire. The instrument comprises five sections: participants select five personally relevant themes from a predefined list of 12 and rate each for importance, well-being, and the impact of both their chronic condition and its treatments. Three scores were generated: Well-being, Health Problem Impact, and Treatment Impact. Psychometric evaluation included face, structural (confirmatory factor analysis), and convergent validity (correlations with SF-36 scores), as well as reliability (Cronbach’s α and McDonald’s ω).

**Results:**

A total of 535 participants (73% women; 27% with chronic illness; aged 18–90 years) completed the study. Most (81%) fully understood the questionnaire, and 96% completed it in under five minutes. The Well-being and Health Problem Impact scores showed satisfactory model fit and reliability (α/ω = 0.66–0.80), while the Treatment Impact score performed less well. The first two scores correlated moderately (*r* > 0.40) with relevant SF-36 dimensions.

**Conclusion:**

The PerQolateur is the first validated, personalized HRQoL tool in French, offering brevity, usability, and clinical relevance for patient-centered care.

**Supplementary information:**

The online version contains supplementary material available at 10.1186/s41687-026-01102-4.

## Key points

-HRQoL questionnaires are widely used in research but remain underused in clinical practice, partly due to their length and lack of personalization

-This study aimed at creating an innovative and personalized HRQoL questionnaire to be quick for the patient to complete (4 items) and easy for clinicians to interpret

-The results showed excellent usability and satisfactory validity and reliability, supporting its suitability for routine clinical use

**Supplementary information:**

The online version contains supplementary material available at 10.1186/s41687-026-01102-4.

## Context

The concept of “quality of life” (QoL) is widely applied across multiple disciplines, particularly in healthcare. The World Health Organization (WHO) defined QoL as *“individuals’ perception of their position in life, in the context of the culture and value systems in which they live, and in relation to their goals, expectations, standards, and concerns. It is a broad-ranging concept, affected in a complex way by physical health, psychological state, personal beliefs, social relationships, and their relationship to salient features of the environment”* [1].* Within this framework,* researchers and clinicians often focus more specifically on “health-related quality of life” (HRQoL), generally understood as a multidimensional construct encompassing various aspects of health (e.g., physical, mental, social, and functional) that influence overall well-being and daily life. HRQoL can be assessed either cross-sectionally (snapshot assessment) or longitudinally to capture changes over time [2]. However, the terms QoL and HRQoL are frequently used interchangeably and require clarification. One alternative is to define HRQoL as the evaluation of how health affects overall quality of life [3].

Unlike health status indicators, which can be partially assessed by healthcare professionals, HRQoL generally requires direct measurement from the patient’s perspective. However, in certain situations—such as cognitive impairment—or within specific populations, such as children, proxy reporting may be necessary. As a form of Patient-Reported Outcome Measures (PROMs), HRQoL is typically assessed using self-administered questionnaires covering multiple dimensions. Both generic or disease-specific tools have proliferated in recent decades. Widely used generic instrument include the SF36, WHOQOL and PROMIS-29 [4–6]. Numerous disease-specific questionnaires have also been developed, often symptom-focused, such as the EORTC QLQ-C30 for cancer [6], the KDQOL for renal failure [7], and the Saint Georges Respiratory questionnaire (SGRQ) for chronic respiratory diseases, in particularly COPD [8].

Despite their value, PROM-based HRQoL scores can be challenging for both clinicians and patients to interpret, even when they are straightforward to compute. Questionnaires may fail to capture domains that are relevant to individual patients, and clinicians may struggle to translate complex results derived from multidimensional instruments into actionable insights for personalized care. Conversely, some instruments relying on standardized scoring systems, although potentially more time-consuming to calculate, are inherently easier to interpret. In addition, severity thresholds have been established for certain instruments, facilitating score interpretation. However, the application of such thresholds to HRQoL measures is generally less appropriate. In addition, these questionnaires are often lengthy (typically 25–50 items), with responses aggregated into validated scores. Computerized Adaptive Tests (CAT) which tailor questions to patients responses, have been proposed to shorten assessments, but their implementation in routine care remains limited due to technological, organizational, and cultural barriers [9]. As a result, although HRQoL PROMs are widely used in clinical research, they remain underutilized in routine clinical practice for several reasons. These include, on the one hand, the limitations outlined above, and on the other, organizational constraints—such as limited consultation time and insufficient personnel to support HRQoL assessment—as well as a predominant clinical focus on symptoms rather than their broader impact. [10, 11].

A qualitative study conducted by our team in 2018 confirmed these challenges: 3 focus groups involving 21 clinicians identified several barriers to the practical use of HRQoL assessments, such as insufficient theoretical knowledge, lack of standardized methods for daily use, and organizational time constraints [11].

More broadly, the literature emphasized that QOL is inherently subjective and highly context-dependent, with individuals best positioned to evaluate their own living conditions [12, 13]. Accordingly, assessments should allow patients to identify domains that matter most to them, evaluate their status in each, and weight their relative importance in shaping overall QoL [14]. To address this need, several “individualized” Qol measurement questionnaires have been developed. The most established are the “Schedule Evaluation of Individual Quality of Life (SEIQol) [15] and the Patient-Generated-Index (PGI) [16]. Others tools such as MYMOP [17] and MACTAR [18] also exist but are less widely used. These instruments do not apply standardized domains for all respondents; instead, they allow individuals to construct their own framework for evaluating Qol.

The SEIQol is administered as a semi-structured interview of about 30 minutes, in which patients identify up to five key life domains, rate their functioning in each on a Visual Analogic Scale, and assign relative weights using a graduated disc. The psychometric properties of this tool are well-established [15], as are those of the French version [19]. The PGI, which focuses on disease consequences, is shorter (about 10 minutes) and requires no additional materials. Patients identify one to five domains, rate their impact over the past week using a 7-point Likert scale, and weight their relative importance using 10 tokens. Although typically interview-based, the PGI can also be self-administered, and a validated French version exists [20]. In clinical settings, these individualized measures are often more feasible than standardized ones, as they capture patients’ specific concerns and provide more nuanced insights into change over time [21].

Nevertheless, while individualized disease-agnostic QoL measures are available, to our knowledge there is no short, self-administered questionnaire, designed specifically to assess HRQol, that is, the impact of health on QoL. Such a tool would facilitate both administration and integration into routine practice.

The aim of this study was therefore to develop and validate a concise, French-language, personalized self-administrated HRQoL questionnaire that is quick and easy for patients to complete and straightforward for clinicians to interpret This instrument is hereafter referred to as the PERQOLATEUR questionnaire (PERception of the impact of a disease and its treatments on Quality Of Life (QOL) by the pATiEnt, a partner in their healthcare journey).

## Methods

**Fig. 1:**
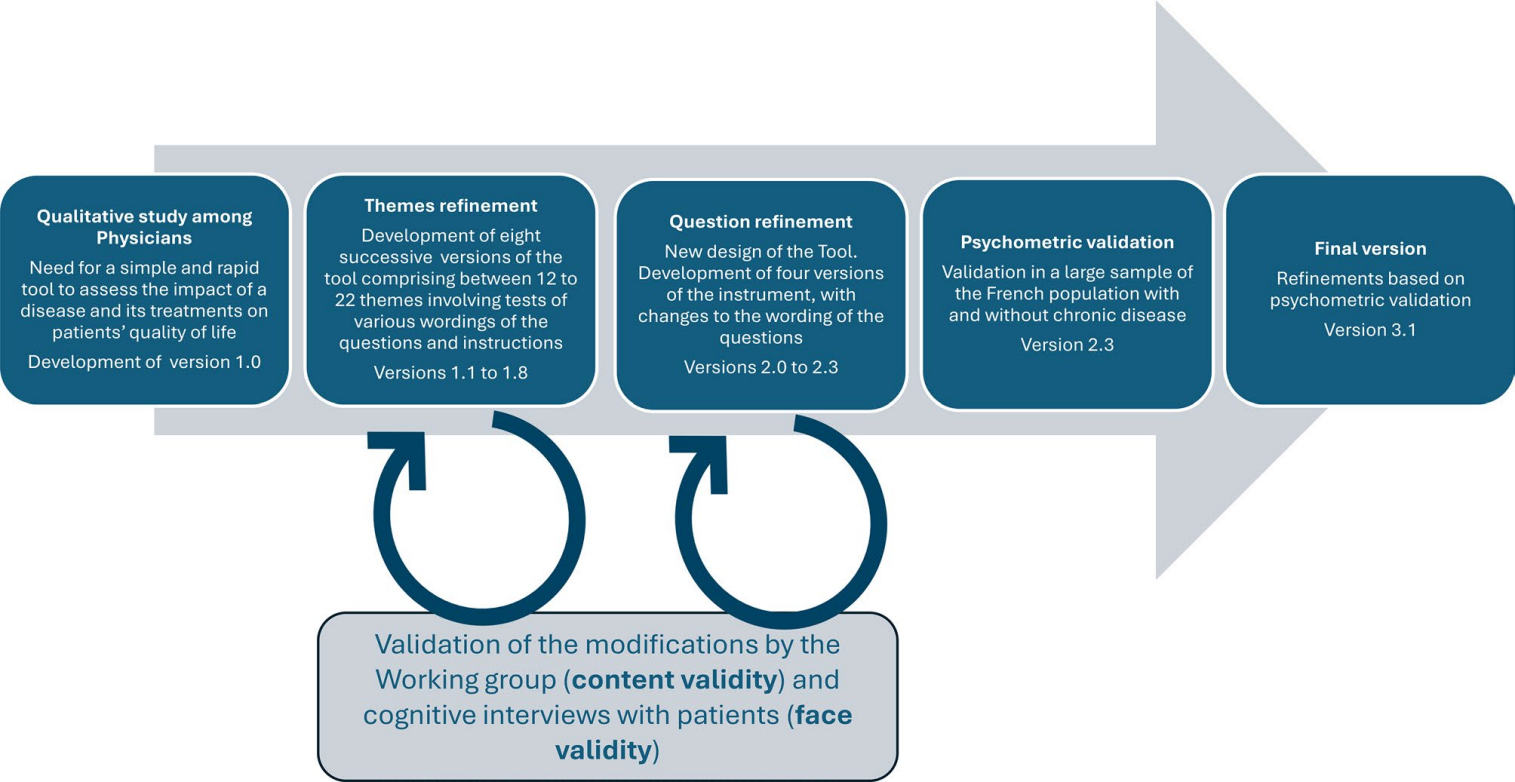
Development and validation process of the questionnaire

The methodology of the development of the PERQOLATEUR questionnaire is illustrated in Fig. 1.

### Design and content validation of the PERQOLATEUR questionnaire

#### Creation of a working group

In 2019, a multidisciplinary research group was established, bringing together researchers in public health, psychometrics, psychology, and health economics. The team also included a practitioner specialist in therapeutic education, a public health physician, public health residents, and two patient partners from “La Fabrique Créative de Santé”, an association dedicated to providing psychosocial support for patients living with chronic illnesses in complement to therapeutic patient education. This group was responsible for developing and validating the content of the questionnaire (relevance and comprehensibility of each item).

#### Questionnaire design methodology

In accordance with the results of the study conducted among clinicians [11], the working group set out to design a self-administered HRQoL questionnaire with the following objectives:

1. To fit on a single A4 page, enabling clinician to view all responses at a glance,
2. To be quick for the patient to complete (under 5 minutes) and easy for clinician to interpret within seconds,
3. To be simple and accessible for patients, avoiding negative or stigmatizing wording.

The tool also needed to allow patients to select the themes most meaningful to them, which could change over time. It was intended to serve both qualitatively, by highlighting key issues rapidly, and quantitatively, by generating easily calculated scores to monitor changes in well-being over time.

#### Tool development

The first step was to generate a list of themes relevant to patients. To do so, the working group drew on the EVAD research study [22], which collected responses from 150 patients with one or more chronic pathologies using the SeiQOL questionnaire to identify and prioritize key themes (unpublished results). A panel of five to eight patients tested each version of the questionnaire. They provided feedback allowing the list to be adjusted and refined. This process led to eight successive versions, with the number of themes evolving substantially from 22 in the most comprehensive version to 12 in the final version, while also including the option for patient to add two personalized themes.

The thematic labels (e.g., “entourage”, “nature”) were intentionally kept broad and were not accompanied by standardized definitions during the development phase. This choice was made to preserve respondents’ subjective interpretation and to allow each individual to anchor these themes in personally meaningful aspects of their life. As quality of life is inherently subjective and context-dependent, participants were encouraged to interpret each theme according to their own experiences and priorities. No formal definitions were provided to respondents during the development or validation phases. While this may introduce some variability in interpretation, it does not affect the scoring, which is based on the relative importance and evaluation of selected domains rather than on strict categorization. This flexible interpretation of themes is consistent with individualized approaches to quality of life assessment [12, 13].

Subsequently, the design of the questionnaire, along with the wording of instructions and items, was refined. The patient panel tested four further versions. This iterative collaboration between experts and patients, aimed at optimizing content and clarity, ensured strong content and face validity in accordance with COSMIN as well as ISPOR recommendations [23–25]. The full development process is illustrated in Fig. 1.

#### Tool structure

Finally, the questionnaire is structured into five sections:

1. Patients select five themes that are most relevant to them.
2. They rate the importance of each selected themes, on a 5-point scale, from 1 (“not very important”) to 5 (“fundamental”).
3. They evaluate their well-being for each selected theme on an 11-point scale, from 0 (“very poor”) to 10 (“excellent”).
4. Patients with chronic conditions then assess the impact of their illness on each selected theme using a 5-point scale, from −2 (“very negative”) to + 2 (“very positive”)
5. Finally, the same patients evaluate the impact of their treatments on each selected theme, also using a 5-point scale, from −2 (“very negative”) to + 2 (“very positive”).

The version used in the present study (2.3) is provided in supplementary materials.

### Validation of the psychometric properties of the PERQOLATEUR tool

The psychometric validation was conducted in accordance with COSMIN guidelines [23].

#### Sample

To recruit a broad and diverse sample, a digital version of the PERQOLATEUR questionnaire was developed. Posters displaying a QR code linking to the questionnaire were displayed in several medical practices (general practitioners, midwives) and within a patient association. Recruitment was further extended via social media (Facebook, Linkedln) targeting the general population. The questionnaire was available online from January 6 to March 9, 2023.

#### Survey questionnaire

The questionnaire consisted of the following sections:

1. Socio-demographic data: age, gender, education level (8 categories), and socio-professional category (11 categories)
2. Presence of a chronic disease and its category (7 categories)
3. PERQOLATEUR questionnaire (complete for people with a chronic illness, those without a chronic illness were exclude from questions on the impact of illness and treatment)
4. EPICES questionnaire comprises 11 questions relatives to material and social precariousness. The EPICES score is the most widely used in the literature and in clinical research in France. It helps identify individuals in precarious situations who do not meet standard socio-administrative criteria. It is psychometrically validated and divided into quintiles, with quintile 1 indicating the absence of precariousness and quintile 5 indicating the highest level of material and social precariousness. A score above 30 combines the last two quintiles and indicates the existence of a high precarious situation [26].
5. Additional section (optional): the SF36 health-related quality of life questionnaire (36 questions) for those who opted to participate further [27]. The SF-36 is a widely used generic HRQoL instrument comprising eight domains: physical functioning, role physical, bodily pain, general health, vitality, social functioning, role emotional, and mental health.

#### Face validity

Face validity was evaluating by measuring the time required to complete the PERQOLATEUR questionnaire and by collecting participants’ feedback regarding its clarity, comprehensibility and perceived usefulness.

#### Scores

Three scores were developed:

1. **General Well-being Score**: Mean of the well-being ratings, weighted by the importance of each theme, on a scale from 0 to 10.
2. **Health Problem Impact Score**: Mean of health problem impact ratings, weighted by importance, ranging from −2 to +2.
3. **Treatment Impact Score**: Mean of treatment impact ratings, weighted by importance, ranging from −2 to +2.

Scores were calculated as weighted means, where each domain score was multiplied by its importance rating. For the well-being score (range 0 to 10), higher values indicate better quality of life. For the impact scores (range −2 to +2), negative values indicate a negative impact and positive values indicate a positive impact.

When data were missing, scores were calculated using all available responses, regardless of the number of selected themes (1 to 5). For individuals without a chronic illness, only the well-being items were answered, allowing calculation of the first score only.

#### Analysis of the relevance of weightings

During construction process of the questionnaire, the working group questioned the relevance of asking respondent to rate the importance of each selected theme (Question 2). To evaluate this weighting system, weighted and unweighted scores were compared. Agreement was quantified using the intraclass correlation coefficient (ICC). The homogeneity of importance ratings was also examined by calculating, for each participant, the difference between their highest and lowest importance ratings. If high concordance (ICC > 0.80) and high homogeneity (≥80% of participants showing a difference ≤ 2) were observed, weighting was considered of limited added value.

#### Construct validity

The structural validity of each score was assessed by testing the fit of each set of questions (well-being, illness impact and treatment impact) to a unidimensional Confirmatory Factor Analysis (CFA) model. Acceptable structural validity was defined as meeting the following thresholds: RMSEA (Root Mean Square Error of Approximation) <0.08, CFI (Comparative Fit Index) >0.9 and TLI (Tucker-Lewis Index) >0.9 [28]. CFAs were performed both on raw items responses and on weighted responses, where the importance ratings were multiplied by the raw item responses.

#### Discriminant validity

It was hypothesized that scores would show little variation across socio-demographic variables (age, gender, level of education). However, a significant difference was expected in the well-being score—the only score calculable for patients without chronic illness—between patients with and without a chronic condition, as well as between the most socioeconomically disadvantaged patients and others. Group comparisons were performed using Student’s t-tests.

#### Convergent validity

Convergent validity was examined through correlation analyses between the three PERQOLATEUR scores and the scores from the generic SF36 questionnaire. Significant correlations were anticipated with each of the SF36 dimensions.

#### Scores’ reliability

The internal consistency reliability of each of the three scores was assessed using Cronbach’s alpha and McDonalds’ Omega [29]. Reliability coefficients above 0.7 were considered acceptable.

## Results

### Sample characteristics

After excluding minors (<18 years − 4 responses), completely empty PERQOLATEUR questionnaires (38 responses) and duplicates (13 responses), 535 unique responses were analysed.

This sample consisted of individuals aged 18 to 90, with 53% under 40, 36% between 40 and 65 and 10% over 65. Women accounted for 73% (389 respondents) and 12% (61 respondents) were in precarious situations, as indicated by the EPICES questionnaire. Additionally, among the respondents, 27% (142 individuals) had a known and declared chronic disease, including 17% with endocrine disease, 13% with heart disease, 12% with lung disease, 12% with mental illness, 9% with digestive disorders, 8% with neurological disease, 5% with cancer and 25% with another types of disease). In terms of education, 32% (173 respondents) had a high school education or less, 19% (102 respondents) held a two- or three-year post-secondary degree, and 49% (260 respondents) had a master’s degree or higher. Regarding occupation, 33% (178 respondents) were senior executives, 23% (120 respondents) were middle managers, employees, or workers, 28% (147 respondents) were students, and 9% (48 respondents) were retirees. Other social categories were underrepresented (7%).

### Face validity and utility of the questionnaire

The PERQOLATEUR questionnaire was fully completed by 480 respondents (90%). No participants reported a complete lack of understanding. 55 patients (11%) felt they understood the questionnaire moderately, 435 (81%) felt they understood it perfectly and 45 (8%) did not answer this question. 67% of patients felt that this questionnaire could be useful for their doctor, 28% considered it somewhat unnecessary, and 5% found it completely irrelevant. The time required completing the socio-demographic questions and the PERQOLATEUR questionnaire was under 5 minutes for 512 respondents (96%).

### Content validity

All 12 themes were selected by respondents (Table 1), with “spiritual life” chosen by a minimum of 11% and “family” chosen by a maximum of 65%. The frequencies for the other themes were as follow: “work” at 56%, “entourage” at 53%, “leisure” at 49%, “healthy living” at 42%, “emotional life” at 41%, “nature” at 34%, “money” and “housing” at 30%, “confidence in the future” at 28% and “children” at 25%. Few new themes were mentioned, including “animals” (mentioned once); “politics” (mentioned twice) and “creativity” (mentioned once).

**Table 1:** Choice of important themes in daily life according to gender, age, precariousness and existence or absence of a chronic illness and results of the discriminant validity

Chosen themes	% of quotation (*n* = 538)	Gender		Age			Precariousness (EPICES score > 30)		Chronic illness	
		Women (*n* = 387)	Men (*n* = 145)	<40 y (*n* = 283)	40–65 y (*n* = 191)	>65y (*n* = 54)	Yes (*n* = 61)	No (*n* = 474)	Yes (*n* = 142)	No (*n* = 393)
Family	65%	66%	63%	65%	64%	70%	69%	64%	59%	66%
Work	56%	58%	49%	69%	53%	4%	64%	54%	39%	61%
Entourage	53%	54%	51%	62%	40%	52%	51%	53%	48%	55%
Leisure	49%	50%	48%	48%	53%	39%	49%	49%	47%	49%
Healthy living	42%	43%	41%	43%	40%	52%	46%	42%	37%	44%
Emotional life	41%	38%	48%	42%	40%	30%	33%	42%	36%	42%
Nature	34%	34%	34%	26%	45%	39%	25%	35%	35%	33%
Money	30%	29%	34%	37%	26%	12%	46%	28%	25%	32%
Housing	30%	30%	30%	32%	30%	24%	43%	28%	34%	29%
Confidence in the future	28%	30%	25%	31%	24%	31%	26%	28%	26%	28%
(Grand) children	25%	26%	23%	17%	28%	63%	20%	26%	25%	25%
Spiritual life	11%	11%	10%	8%	12%	22%	18%	10%	13%	9%
Unweighted well-being score (*n* = 504) [Mean±SE] Range 0 to 10	6.6 ± 1.5	6.6 ± 1.5	6.8 ± 1.4	6.5 ± 1.5	6.5 ± 1.5	7.5 ± 1.2	6.7 ± 1.4	5.8 ± 1.6	6.6 ± 1.5	6.6 ± 1.5
		p = 0.082		p < 0.0001			p < 0.001		p = 0.61	
Unweighted impact score of illness on well-being (*n* = 125) [Mean±SE] Range −2 to +2	−0.3±0.5	−0.4±0.6	−0.2±0.4	−0.5±0.5	−0.3±0.5	−0.0±0.4	−0.3±0.5	−0.5±0.7		
		p = 0.081		p < 0.001			p = 0.71			
Unweighted impact score of treatment on well-being (*n* = 125) [Mean±SE] Range −2 to +2	+0.1±0.5	+0.1±0.5	+0.1±0.4	+0.1±0.5	+0.1±0.4	+0.2±0.5	+0.1±0.4	+0.2±0.5		
		p = 0.45		p = 0.61			p = 0.45			

### Interest in weighting

The intraclass correlation coefficients (ICCs) showed highly consistent values between the scores weighted by importance and the unweighted scores: ICC = 0.84 for the well-being score, ICC = 0.99 for the health problems’ impact score, and ICC = 0.98 for the treatment impact’ score. Additionally, the weightings were extremely homogeneous: 61% of individuals provided homogeneous scores with a maximum difference of 1 point (out of 5), and 94% with a maximum difference of 2 points (out of 5).

### Psychometric validation of the three scores

For each of the 3 scores, the indices for structural validity, and internal consistency reliability are summarized in Table 2.

**Table 2:** Structural validity and internal consistency reliability indices for the 3 scores

Scores Indices	Well-being Score (*n* = 504)	**Health Problems impact Score** (*n* = 125)	**Treatment impact Score** (*n* = 125)
Structural validity			
X^2^ (5 df)	12.47	7.85	15.53
RMSEA	0.056	0.070	0.127
CFI	0.974	0.976	0.942
TLI	0.948	0.952	0.883
Reliability			
Cronbach’s alpha	0.66	0.75	0.79
McDonald’s omega	0.67	0.75	0.80

For structural validity, the CFA model fit was satisfactory without adding any correlation between residuals for Well-being and Health-problems impact scores.

Reliability was higher for Health-problems and Treatment impact scores than for Well-being score.

Discriminant validity (Table 1) revealed significant differences between age groups across all three scores, and, surprisingly, a significantly higher well-being score among individuals in precarious situations. In contrast, no significant difference existed between individuals with and without a chronic illness.

Concerning the convergent validity (Table 3), the “Well-being” and “Health Problems Impact” scores were positively correlated (*r* = 0.40), indicating that the presence of a health problem can affect well-being. The well-being score is positively correlated (*r* > 0.40) with the “Vitality,” “Social Functioning,” and “Mental Health” dimensions of the SF-36, but less correlated with other dimensions (mainly physical ones). The “Health Problems Impact” score is positively correlated with the same dimensions, as well as the “Emotional Role” and “General Health” dimensions. The “Treatment Impact” score is only weakly correlated with the SF-36 dimensions.

**Table 3:** Convergent validity: correlation coefficient between PERQOLATEUR scores and SF-36 scores

		PERQOLATEUR questionnaire		
		Score 1	Score 2	Score 3
PERQOLATEUR questionnaire	**Score 1 -** Well-being score	1.00	**0.45**	0.10
	**Score 2 -** Health problems impact score		1.00	0.31
	**Score 3 -** Treatment impact score			1.00
SF36 questionnaire	**PF -** Physical Functioning	0.08	0.13	0.03
	**RP -** Role Physical	0.25	0.37	0.11
	**BP -** Body Pain	0.23	0.13	0.09
	**GH -** General Health	0.34	**0.41**	0.11
	**VT -** Vitality	**0.48**	**0.43**	0.16
	**SF -** Social Functioning	**0.52**	**0.46**	0.09
	**RE -** Role Emotional	0.39	**0.40**	−0.04
	**MH -** Mental Health	**0.56**	**0.40**	0.02
	**PCS -** Physical Composite Score	0.02	0.13	0.13
	**MCS -** Mental Composite Score	**0.56**	**0.46**	0.00

## Discussion

Through a rigorous development process involving a multi-professional project team and a panel of patients, the PERQOLATEUR questionnaire was designed to meet its initial specifications: a self-assessment disease-agnostic HRQoL questionnaire, that fit on a single A4 page, is quick to complete (average completion time of 5 minutes), and is easy to understand (more than 80% of respondents reported perfect comprehension). Content validity was carefully established to capture well-being across 12 key life domains. Face validity was subsequently confirmed through qualitative patients’ interviews, and quantitative feedback from a diverse sample regarding clarity, acceptability and completion time. And so, the present study provides initial evidence supporting the validity and internal-consistency reliability of the PERQOLATEUR questionnaire. Psychometric validation on a large sample confirmed the robustness of the tool, while also revealing that the importance-rating question (item 2) did not provided additional relevant information. Weighted and unweighted scores showed high concordance (ICC > 0.8), and importance ratings were highly homogeneous across respondents. Consequently, the weighting system was deemed unnecessary, as it did not meaningfully impact the score, and Item 2 was removed.

The PERQOLATEUR questionnaire ultimately measures three meaningful dimensions: global well-being, the impact of health problems, and the impact of treatments on the patient’s well-being. Psychometric properties were satisfactory for the first two scores, while the treatment impact score was weaker. This may reflect greater heterogeneity in patients’ perceptions of treatment effects, which can include both positive and negative aspects and may be less directly experienced than the impact of the disease itself. The internal consistency reliability of the well-being score was lower than that of the other scores. This may be partly explained by the individualized nature of the questionnaire, as respondents select different domains, which can reduce inter-item homogeneity. While this may be considered as a limitation from a classical psychometric perspective, it is also inherent to individualized approaches to quality of life assessment, which prioritize relevance to the individual over uniformity across respondents.

Structural validity was adequate, although some unexpected results emerged. Older participants (>65 years) reported higher well-being scores than younger respondents, and socioeconomically disadvantaged participantsalso reported higher well-being scores than their peers. These paradoxical results may reflect a “healthy volunteer effect” bias: individuals with good quality of life and strong social networks may have been more inclined to participate, while those with poorer QoL may have been underrepresented. This interpretation is consistent with the relatively high education levels observed in the sample compared with the general population.

Several limitations should be acknowledged. The sample was subjected to selection bias, with underrepresentation of older adults, a limited proportion of participants with chronic diseases (one-third), and overrepresentation of individuals with higher socio-economic status. These characteristics may influence QoL perceptions and limit the generalizability of the findings, particularly for the assessment of chronic diseases’ impact. While strict representativeness is not a prerequisite for psychometric validation, which primarily requires sufficient sample size and response variability, the composition of the sample remains an important consideration. Further studies are therefore needed to validate the questionnaire in more diverse and clinically representative populations [30].

The individualized and disease-agnostic nature of the questionnaire also entails several methodological challenges. As respondents select different domains, scores may not be directly comparable across individuals, as they may reflect different underlying aspects of quality of life. This limits the interpretability of aggregated results at the group level. In addition, the interpretation of changes over time may be affected by response shift, as patients may modify the domains they consider most important. In such cases, variations in scores may reflect both changes in perceived well-being and changes in priorities.

Finally, this variability in domain selection may influence certain psychometric properties, as the content underlying the scores is not fixed across respondents or over time. These limitations are inherent to individualized approaches to QoL assessment and should be considered when interpreting results, particularly in research settings.

The tool was validated in its digital format, which offers advantages in usability: once themes are selected, subsequent questions are automatically directed to those themes. In contrast, the paper version requires respondents to follow a more complex structure, although the qualitative study suggests that this was well understood by patients. Previous studies have shown that, when faithfully migrated, electronic and paper versions of patient-reported outcome measures demonstrate comparable psychometric properties [31]. As a result, a computerized version tailored for clinical use, allowing automatic score calculation, is under development.

The final version of the PERQOLATEUR questionnaire (v3.1) comprises four items that are concise, simple and user-friendly. It represents an advance over existing individualized measures by removing the need for weighting procedures or interviewer involvement. It provides evidence of validity and internal-consistency reliability and appears to be a promisinginstrument for assessing individualized HRQoL in clinical practice for French people. Further studies are needed to assess additional psychometric properties, including test–retest reliability and responsiveness to change. Consequently, a research study protocol has just been submitted to test the questionnaire on a group of patients participating in a therapeutic education program for a chronic condition. On the one hand, this project will examine the instrument’s implementability in clinical practice by healthcare professionals and patients (acceptability, feasibility, appropriateness, and ability to promote shared decision-making). On the other hand, it will test new psychometric properties, such as responsiveness to change.

Both the validated version (v2.3) and the updated version (v3.1) and their user manuals are available in the supplementary materials, in French and in English translation (the latter pending validation).

-This study aimed at creating an innovative and personalized HRQoL questionnaire to be quick for the patient to complete (4 items) and easy for clinicians to interpret

-The results showed excellent usability and satisfactory validity and reliability, supporting its suitability for routine clinical use

## Electronic supplementary material

Below is the link to the electronic supplementary material.

Supplementary Material 1

Supplementary Material 2

Supplementary Material 3

Supplementary Material 4

Supplementary Material 5

## Data Availability

The datasets generated analysed during the current study are available in the following repository: 10.57745/VGZHRX.

## Abbreviations

QoL: Quality of life
HRQoL: Health Related Quality of Life
PROM: Patient Related Outcome Measure
SEIQol: Schedule Evaluation of Individual Quality of Life
PGI: Patient-Generated-Index
CFA: Confirmatory Factor Analysis
RMSEA: Root Mean Square Error of Approximation
CFI: Comparative Fit Index
TLI: Tucker-Lewis Index
ICC: Intraclass Correlation Coefficient
