# Supplementary material for: Development and psychometric validation of the PERQOLATEUR questionnaire: an individualized self-report measure of the impact of chronic illness and treatment in clinical practice
Source: J Patient Rep Outcomes. 2026 Jun 9;10:145. doi: 10.1186/s41687-026-01102-4 (PMC13481634; doi:10.1186/s41687-026-01102-4)
Supplement: Supplementary file 2 [file 41687_2026_1102_MOESM2_ESM.pdf]

Impact of your health problems and treatments on daily life

SPHERE methodS in Patient-centered outcomes & HEalth ResEarch

Inserm

1) Here is a list of daily life themes.

- **A.** Social circle
- **B.** Money
- **C.** Romantic life
- **D.** Leisure/Relaxation/ Little Pleasures
- **E.** (Grand)children
- **F.** Family
- **G.** Nature
- **H.** Housing
- **I.** Confidence in the future
- **J.** Lifestyle hygiene
- **K.** Work
- **L.** Spiritual life
- **M.** Other: ....
- **N.** Other: ....

If you could only choose 5, which themes do you think are the most important in your life right now?
Write the corresponding letters in the boxes below.

......	......	......	......	......

2) What is, for you today, **your level of well-being** for each of the themes?
*Give a value **between 0** (=very poor) **and 10** (=excellent).*

......	......	......	......	......

3) Do your health problem(s) have an impact on these important themes in your life?
Check the corresponding icon for each theme (☹ *= very negative,* 🙁 *= negative,* 😐 *= neutral,* 🙂 *= positive,* ☺ *= very positive)*

☹ 🙁 😐 🙂 ☺	☹ 🙁 😐 🙂 ☺	☹ 🙁 😐 🙂 ☺	☹ 🙁 😐 🙂 ☺	☹ 🙁 😐 🙂 ☺
☐ ☐ ☐ ☐ ☐	☐ ☐ ☐ ☐ ☐	☐ ☐ ☐ ☐ ☐	☐ ☐ ☐ ☐ ☐	☐ ☐ ☐ ☐ ☐

4) Do your treatments have an impact on these important themes in your life?
Check the corresponding icon for each theme (☹ *= very negative,* 🙁 *= negative,* 😐 *= neutral,* 🙂 *= positive,* ☺ *= very positive)*

☹ 🙁 😐 🙂 ☺	☹ 🙁 😐 🙂 ☺	☹ 🙁 😐 🙂 ☺	☹ 🙁 😐 🙂 ☺	☹ 🙁 😐 🙂 ☺
☐ ☐ ☐ ☐ ☐	☐ ☐ ☐ ☐ ☐	☐ ☐ ☐ ☐ ☐	☐ ☐ ☐ ☐ ☐	☐ ☐ ☐ ☐ ☐

Authors: Jean-Benoit Hardouin, Leila Moret
perqolateur@univ-nantes.fr

PerQolateur questionnaire
version 3.1 - September 2025
Non-validated version

CC BY NC ND
UMR INSERM 1246 - SPHERE
icon made by Freepik from Flaticon
