# Supplementary material for: Development and psychometric validation of the PERQOLATEUR questionnaire: an individualized self-report measure of the impact of chronic illness and treatment in clinical practice
Source: J Patient Rep Outcomes. 2026 Jun 9;10:145. doi: 10.1186/s41687-026-01102-4 (PMC13481634; doi:10.1186/s41687-026-01102-4)
Supplement: Supplementary file 3 [file 41687_2026_1102_MOESM3_ESM.pdf]

Impact de vos problèmes de santé et de vos traitements sur la vie quotidienne

SPHERE methodS in Patient-centered outcomes & HEalth ResEarch

Inserm

1) Voici une liste de thèmes de la vie quotidienne

**A.** Entourage	**E.** (Petits) enfants	**I.** Confiance en l'avenir
**B.** Argent	**F.** Famille	**J.** Hygiène de vie
**C.** Vie sentimentale	**G.** Nature	**K.** Travail
**D.** Loisirs/Détente/ Petits Plaisirs	**H.** Logement	**L.** Vie spirituelle

M. Autre : ....

N. Autre : ....

Si vous ne deviez en choisir que 5, quels seraient les thèmes qui vous semblent le plus important dans votre vie actuellement ? *Reportez les lettres correspondantes dans les cases ci dessous*

......	......	......	......	......

2) Quel est, aujourd'hui, pour vous, **votre niveau de bien-être** pour chacun des thèmes ?
*Donnez une valeur **entre 0** (=très mauvais) **et 10** (=excellent)*

......	......	......	......	......

3) Votre/vos problème(s) de santé ont-ils un impact sur ces thèmes importants de votre vie ?
Cochez le pictogramme correspondant pour chaque thème (☹ = très négatif, = négatif, = neutre, = positif, ☺ = très positif)

☹ ☐	☐	☐	☐	☺ ☐

4) Vos traitements ont-ils un impact sur ces thèmes importants de votre vie ?
Cochez le pictogramme correspondant pour chaque thème (☹ = très négatif, = négatif, = neutre, = positif, ☺ = très positif)

☹ ☐	☐	☐	☐	☺ ☐

Auteurs : Jean-Benoit Hardouin, Leila Moret
perqolateur@univ-nantes.fr

Questionnaire PerQolateur
version 3.1 - Septembre 2025
Version validée

CC BY NC ND UMR INSERM 1246 - SPHERE
icon made by Freepik from Flaticon
