# Supplementary material for: Development and psychometric validation of the PERQOLATEUR questionnaire: an individualized self-report measure of the impact of chronic illness and treatment in clinical practice
Source: J Patient Rep Outcomes. 2026 Jun 9;10:145. doi: 10.1186/s41687-026-01102-4 (PMC13481634; doi:10.1186/s41687-026-01102-4)
Supplement: Supplementary file 4 [file 41687_2026_1102_MOESM4_ESM.pdf]

PERQOLATEUR QUESTIONNAIRE – USER MANUAL

Editor: Jean-Benoit Hardouin

Date: September 25, 2025

PerQoLateur Questionnaire Version: 3.1 (September 2025)

PerQolateur: PERception of the impact of a disease and its treatments on Quality Of Life (QOL) by the pATiEnt, a partner in their healthcare joURney

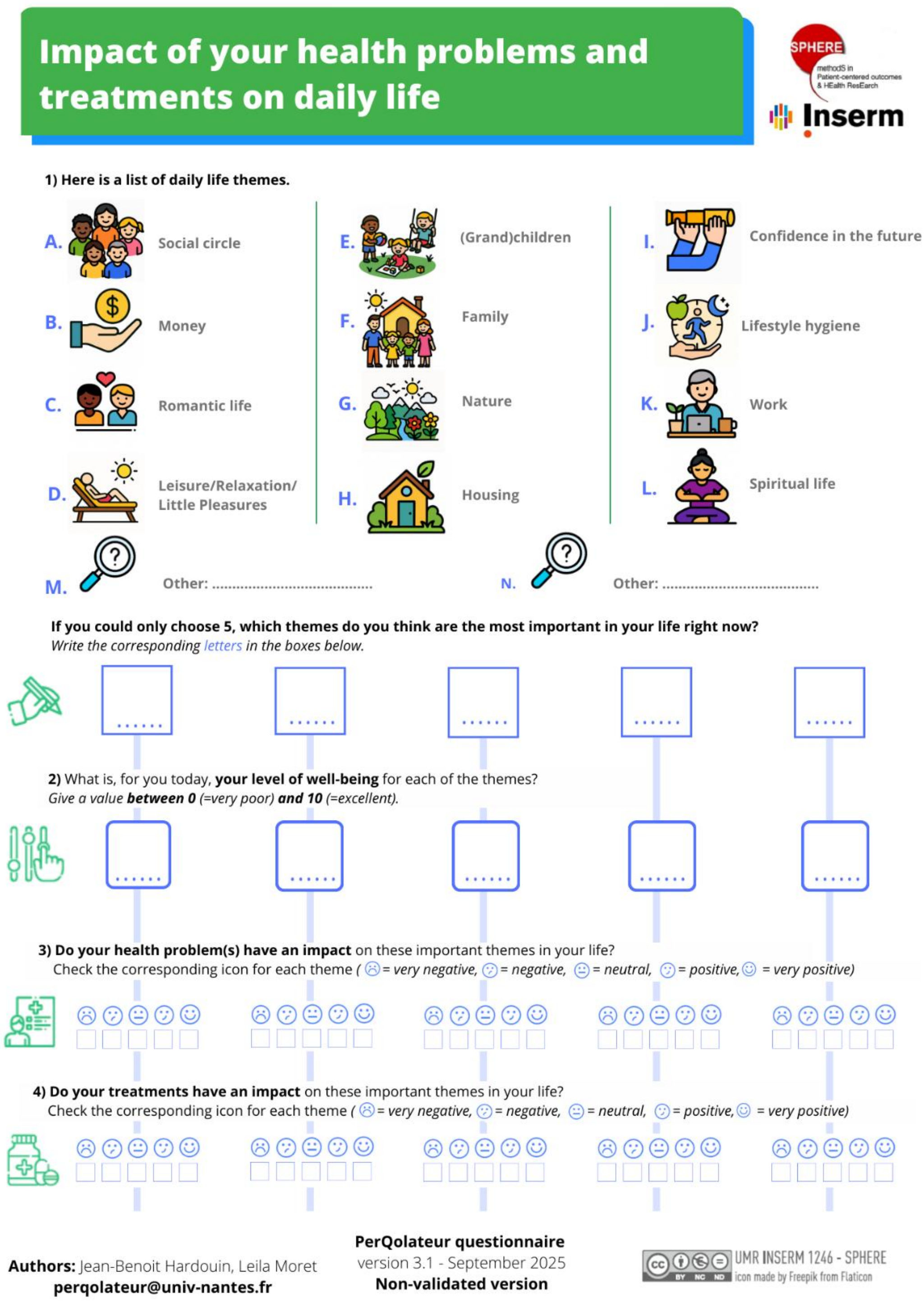

Impact of your health problems and treatments on daily life

SPHERE methodS in Patient-centered outcomes & HEalth ResEarch

Inserm

1) Here is a list of daily life themes.

A. Social circle
B. Money
C. Romantic life
D. Leisure/Relaxation/ Little Pleasures
E. (Grand)children
F. Family
G. Nature
H. Housing
I. Confidence in the future
J. Lifestyle hygiene
K. Work
L. Spiritual life
M. Other: ....
N. Other: ....

If you could only choose 5, which themes do you think are the most important in your life right now?
Write the corresponding letters in the boxes below.

....

2) What is, for you today, **your level of well-being** for each of the themes?
Give a value ***between 0*** *(=very poor)* ***and 10*** *(=excellent).*

....

3) Do your health problem(s) have an impact on these important themes in your life?
Check the corresponding icon for each theme (*= very negative,* *= negative,* *= neutral,* *= positive,* *= very positive)*

4) Do your treatments have an impact on these important themes in your life?
Check the corresponding icon for each theme (*= very negative,* *= negative,* *= neutral,* *= positive,* *= very positive)*

Authors: Jean-Benoit Hardouin, Leila Moret
perqolateur@univ-nantes.fr

PerQolateur questionnaire
version 3.1 - September 2025
Non-validated version

UMR INSERM 1246 - SPHERE
icon made by Freepik from Flaticon

Objective of the PerQoLateur Questionnaire: To assess the impact of a (chronic) illness and its treatments on quality of life themes that are important to the patient.

Administration mode of the PerQolateur questionnaire: The PerQolateur questionnaire is a self-administered (self-evaluation) instrument, meaning that it is designed to be completed independently by the patient, without interaction with a healthcare professional or a relative. It is currently available in paper format only.

USE OF THE QUESTIONNAIRE

1. **The patient selects 5 themes that are important to them, either from the 12 themes provided (A to L), or from 2 additional themes of their choice (M and N).** The patient reports the corresponding letter in response to question 1.

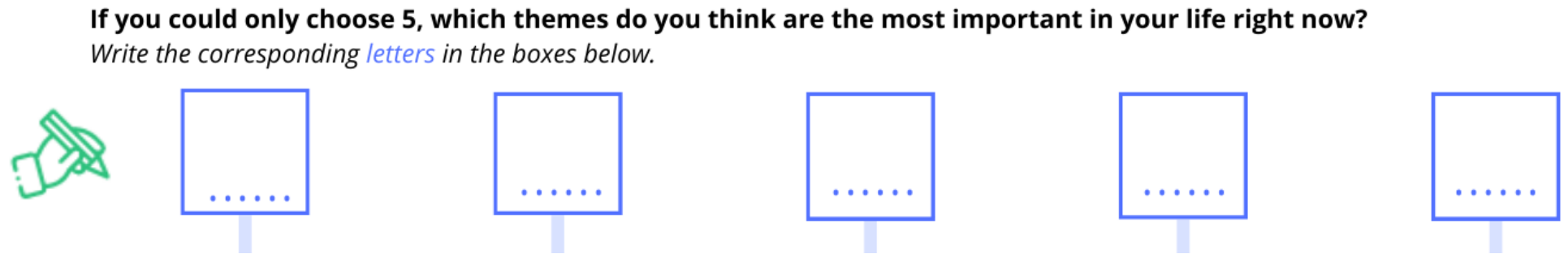
If you could only choose 5, which themes do you think are the most important in your life right now?
Write the corresponding letters in the boxes below.

2. **The patient then indicates their level of well-being in each theme, from 0 (very poor) to 10 (excellent).** The 5 ratings are independent of each other.

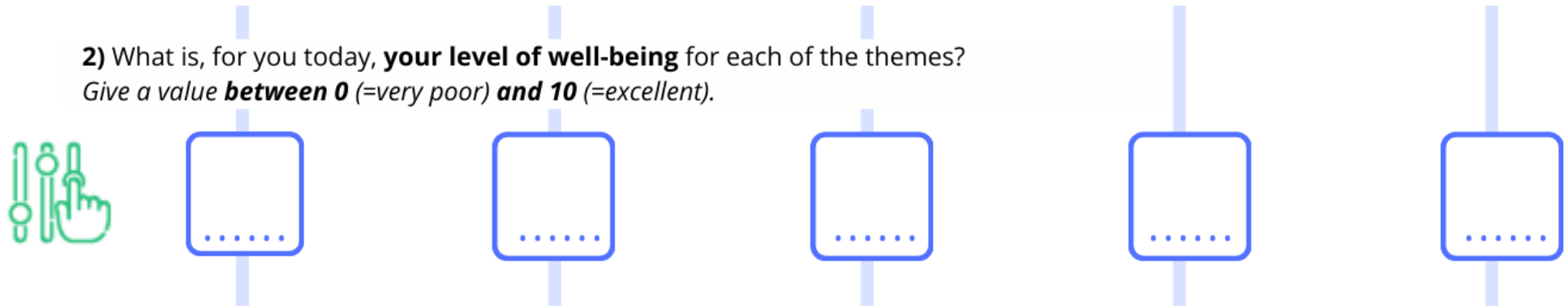
2) What is, for you today, **your level of well-being** for each of the themes?
Give a value ***between 0*** *(=very poor)* ***and 10*** *(=excellent).*

3. **The patient then indicates their perception of the impact of their (chronic) illness on each theme using 4 smileys (from very negative to somewhat positive).** This question should be skipped if the patient does not have a chronic illness.

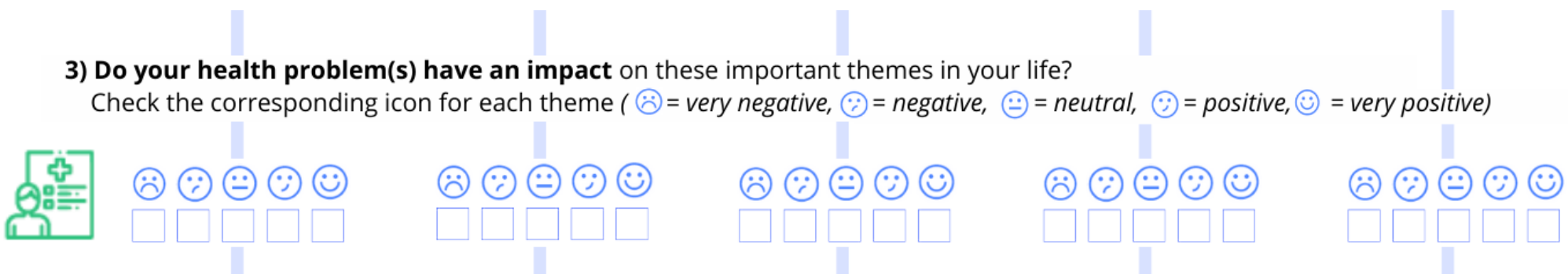
3) Do your health problem(s) have an impact on these important themes in your life?
Check the corresponding icon for each theme *(☹ = very negative, 🙁 = negative, 😐 = neutral, 🙂 = positive, ☺ = very positive)*

4. **Finally, the patient indicates their perception of the impact of their treatments on each theme using 4 smileys (from somewhat negative to very positive).** This question should be skipped if the patient does not have a chronic illness or treatment for a chronic illness.

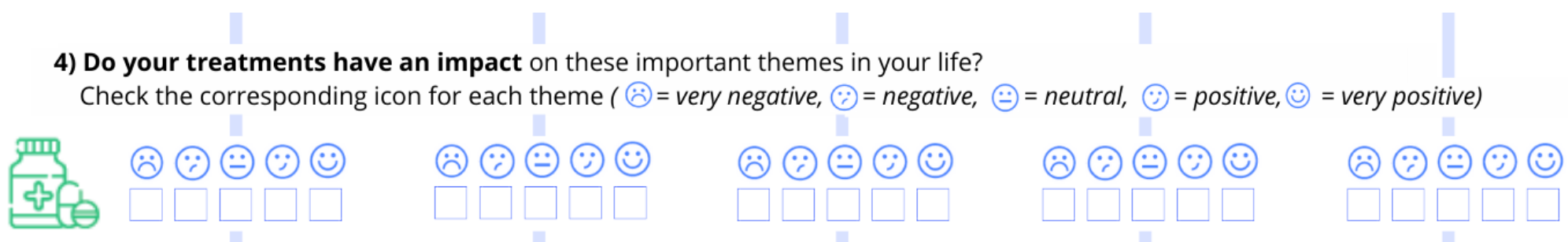
4) Do your treatments have an impact on these important themes in your life?
Check the corresponding icon for each theme *(☹ = very negative, 🙁 = negative, 😐 = neutral, 🙂 = positive, ☺ = very positive)*

QUALITATIVE USE OF THE QUESTIONNAIRE

The PerQoLateur questionnaire was initially designed for qualitative use. In this context, the idea was primarily for the clinician who follows patients **to identify the themes negatively impacted** (☹ 🙁) by the illness and/or treatments in order to modify therapeutic management. This allowed the clinician to initiate a conversation with the patient to better understand their negative feelings, without getting lost in the broad concept of quality of life, where the clinician might feel overwhelmed or unsure of how to act.

QUANTITATIVE USE OF THE QUESTIONNAIRE

A QUANTITATIVE USE OF THE PERQOLATOR QUESTIONNAIRE WITH 3 SCORES:

- The well-being score
- The disease impact score
- The treatment impact score

THE WELL-BEING SCORE

It is calculated by taking the average of the responses to question 2.

$$Score_{Well-being} = \frac{\sum Q2}{n}$$

It is calculated by taking the average of the well-being responses (Q2) for the n themes filled out by the patient (normally 5). The score ranges from 0 to 10.

THE HEALTH PROBLEMS IMPACT SCORE

It is calculated by taking the average of the responses to question 3.

$$Score_{Health\_Problems\_impact} = \frac{\sum Q3}{n}$$

With n being the number of themes filled out by the patient (normally 5) and Q3 being the responses to the disease impact for the n themes, using the following codes: very negative = -2, negative = -1, neutral = 0, somewhat positive = 1, and very positive = 2. The score ranges from -2 to 2. Negative scores represent negative impacts of the disease, while positive scores represent positive impacts of the disease.

THE TREATMENT IMPACT SCORE

It is calculated by taking the average of the responses to question 4.

$$Score_{Treatment\_impact} = \frac{\sum Q4}{n}$$

With n being the number of themes filled out by the patient (normally 5) and Q4 being the responses to the treatment impact for the n themes, using the following codes: very negative = -2, negative = -1, neutral = 0, somewhat positive = 1, and very positive = 2. The score ranges from -2 to 2. Negative scores represent negative impacts of the treatments, while positive scores represent positive impacts of the treatments.

EXAMPLE

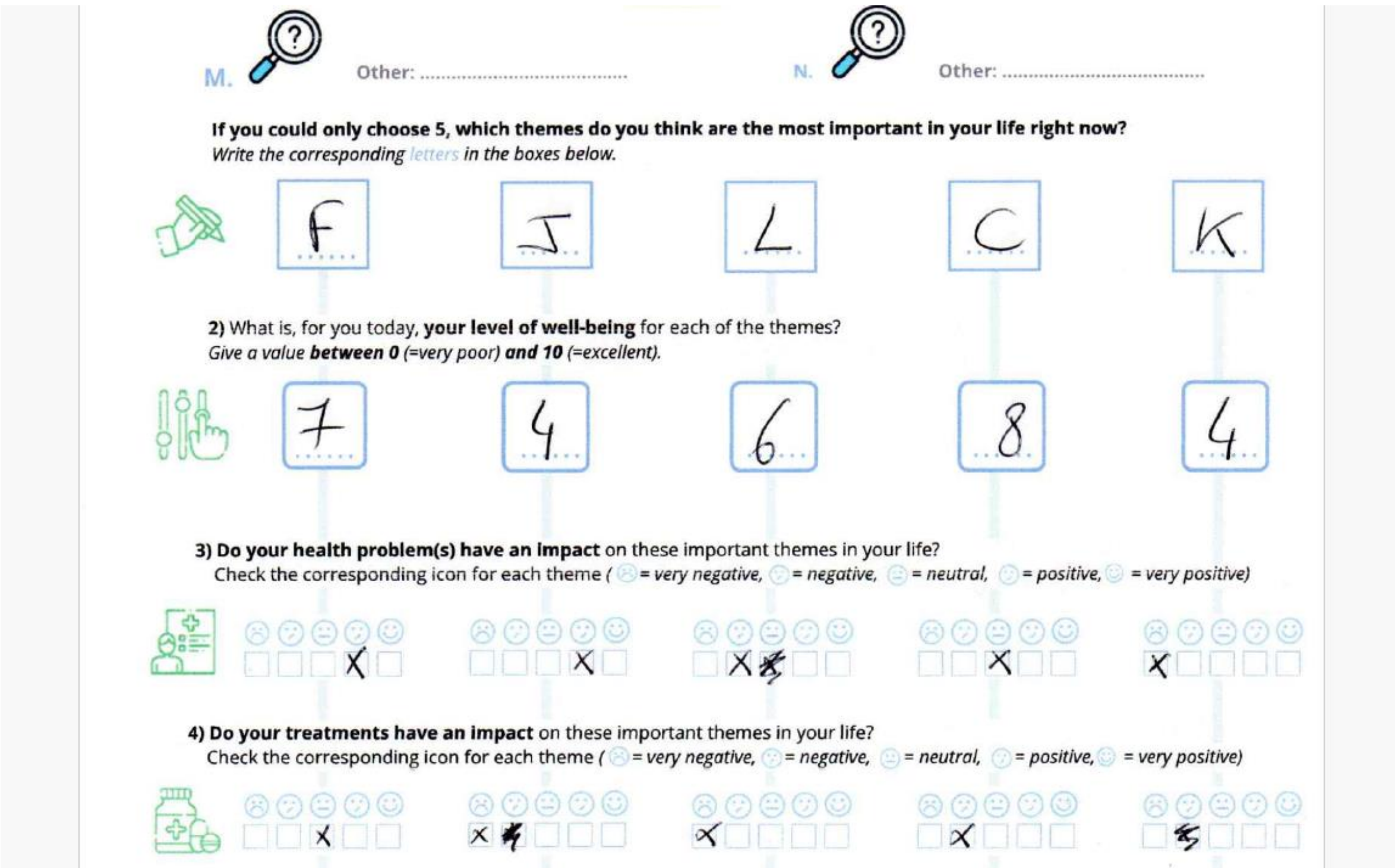

M. Other: .... N. Other: ....

If you could only choose 5, which themes do you think are the most important in your life right now?
Write the corresponding letters in the boxes below.

F	J	L	C	K

2) What is, for you today, **your level of well-being** for each of the themes?
*Give a value **between 0** (=very poor) **and 10** (=excellent).*

7	4	6	8	4

3) Do your health problem(s) have an impact on these important themes in your life?
Check the corresponding icon for each theme (*= very negative,* *= negative,* *= neutral,* *= positive,* *= very positive)*

4) Do your treatments have an impact on these important themes in your life?
Check the corresponding icon for each theme (*= very negative,* *= negative,* *= neutral,* *= positive,* *= very positive)*

$$Score_{Well-being} = \frac{7+4+6+8+4}{5} = \frac{29}{5} = 5.80$$

$$Score_{Health\_problems\_impact} = \frac{1+1+(-1)+0+(-2)}{5} = \frac{-1}{5} = -0.20$$

$$Score_{Treatment\_impact} = \frac{0+(-2)+(-2)+(-1)}{4} = \frac{-5}{4} = -1.25$$
