# Supplementary material for: Development and psychometric validation of the PERQOLATEUR questionnaire: an individualized self-report measure of the impact of chronic illness and treatment in clinical practice
Source: J Patient Rep Outcomes. 2026 Jun 9;10:145. doi: 10.1186/s41687-026-01102-4 (PMC13481634; doi:10.1186/s41687-026-01102-4)
Supplement: Supplementary file 5 [file 41687_2026_1102_MOESM5_ESM.pdf]

QUESTIONNAIRE PERQOLATEUR – MANUEL D’UTILISATION

Rédacteur : Jean-Benoit Hardouin

Date : 25 septembre 2025

Version du questionnaire PerQolateur : 3.1 (septembre 2025)

PerQolateur : PERception de l’impact d’une maladie et de ses traitements sur la qualité de vie (QOL) par le pATient, actEUR de son parcours de soin

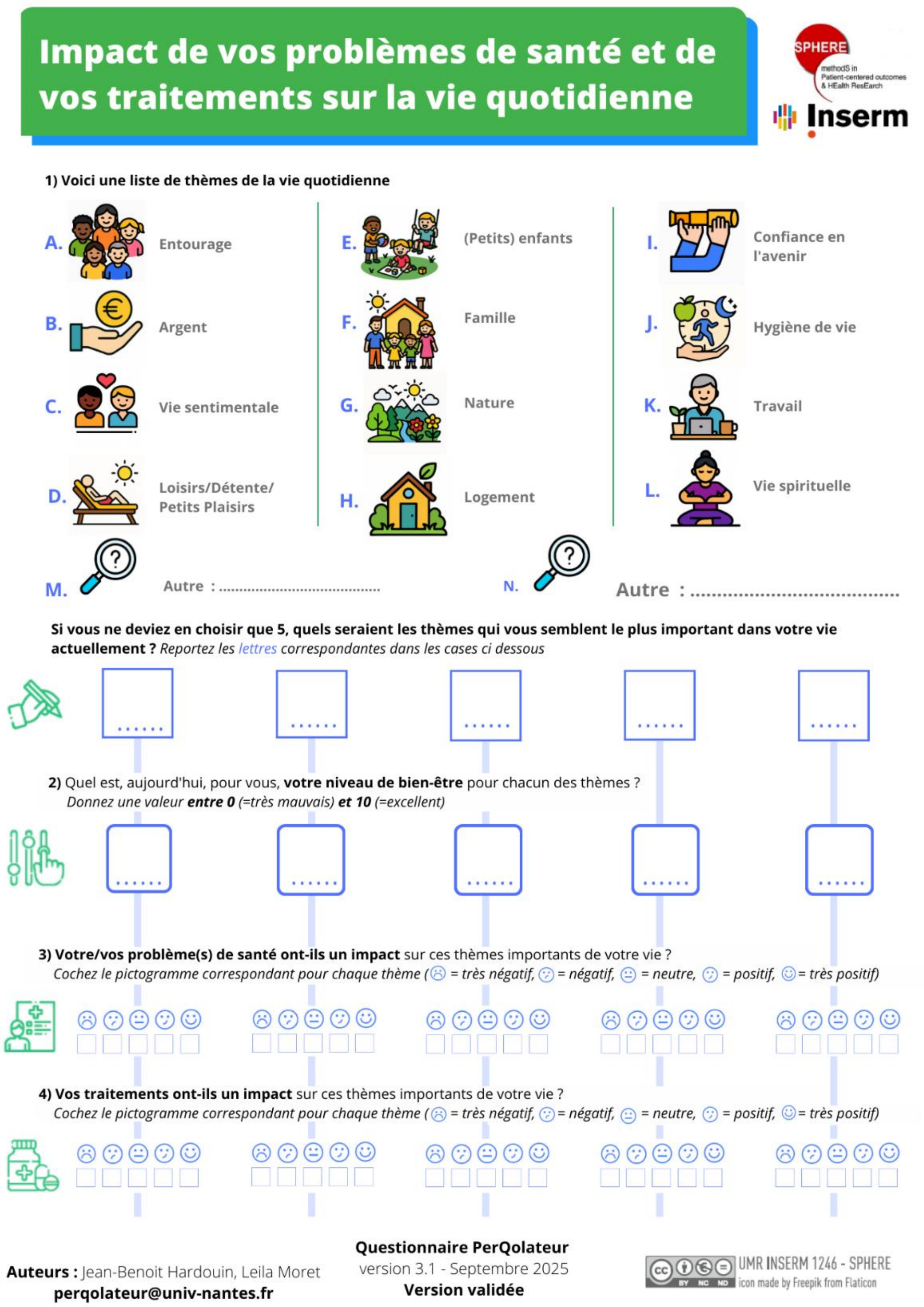

Impact de vos problèmes de santé et de vos traitements sur la vie quotidienne

SPHERE methodS in Patient-centered outcomes & HEalth ResEarch

Inserm

1) Voici une liste de thèmes de la vie quotidienne

A. Entourage
B. Argent
C. Vie sentimentale
D. Loisirs/Détente/ Petits Plaisirs
E. (Petits) enfants
F. Famille
G. Nature
H. Logement
I. Confiance en l'avenir
J. Hygiène de vie
K. Travail
L. Vie spirituelle
M. Autre : ....
N. Autre : ....

Si vous ne deviez en choisir que 5, quels seraient les thèmes qui vous semblent le plus important dans votre vie actuellement ? *Reportez les lettres correspondantes dans les cases ci dessous*

2) Quel est, aujourd'hui, pour vous, **votre niveau de bien-être** pour chacun des thèmes ?
*Donnez une valeur **entre 0** (=très mauvais) **et 10** (=excellent)*

3) Votre/vos problème(s) de santé ont-ils un impact sur ces thèmes importants de votre vie ?
Cochez le pictogramme correspondant pour chaque thème (☹ = très négatif, 🙁 = négatif, 😐 = neutre, 🙂 = positif, ☺ = très positif)

4) Vos traitements ont-ils un impact sur ces thèmes importants de votre vie ?
Cochez le pictogramme correspondant pour chaque thème (☹ = très négatif, 🙁 = négatif, 😐 = neutre, 🙂 = positif, ☺ = très positif)

Auteurs : Jean-Benoit Hardouin, Leila Moret
perqolateur@univ-nantes.fr

Questionnaire PerQolateur
version 3.1 - Septembre 2025
Version validée

UMR INSERM 1246 - SPHERE
icon made by Freepik from Flaticon

Objectif du questionnaire PerQolateur : appréhender l'impact d'une maladie (chronique) et des traitements sur les thématiques de qualité de vie importantes pour le patient

Mode de passation du questionnaire PerQolateur : le questionnaire PerQolateur est un questionnaire en autoadministration (self-evaluation) c'est-à-dire qu'il est prévu pour être rempli en autonomie par le patient, sans interaction avec un soignant ou un proche. Il est actuellement disponible en format papier seulement.

UTILISATION DU QUESTIONNAIRE :

1. **Le patient choisit 5 thèmes importants pour lui, soit parmi les 12 thèmes proposés (de A à L), soit parmi 2 thèmes supplémentaires au choix (M et N).** Il rapporte les lettres correspondantes à la question 1

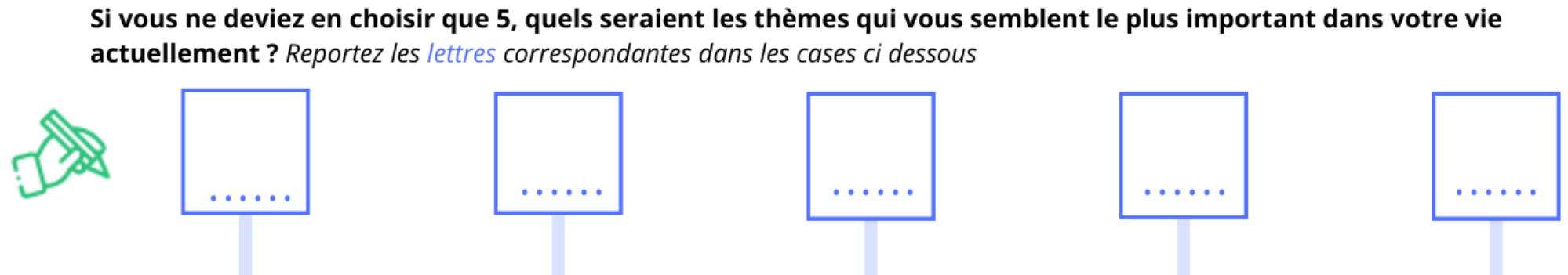

Si vous ne deviez en choisir que 5, quels seraient les thèmes qui vous semblent le plus important dans votre vie actuellement ? *Reportez les lettres correspondantes dans les cases ci dessous*

2. **Il indique alors son niveau de bien être dans chacun des thèmes de 0 (thèse mauvais) à 10 (excellent).** Les 5 cotations sont indépendantes les unes des autres.

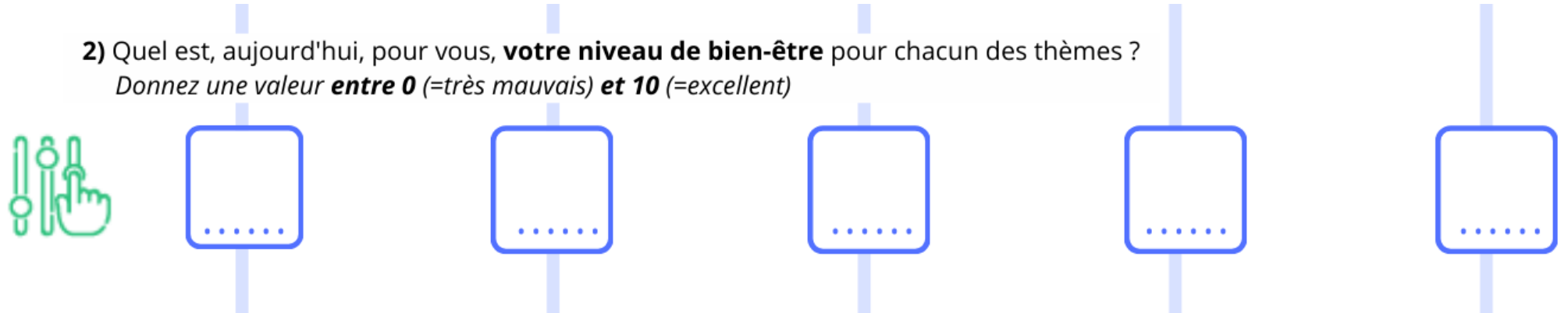

2) Quel est, aujourd'hui, pour vous, **votre niveau de bien-être** pour chacun des thèmes ?
*Donnez une valeur **entre 0** (=très mauvais) **et 10** (=excellent)*

3. **Il indique alors son ressenti de l'impact de sa maladie (chronique) sur chacun des thèmes à l'aide 5 smileys (de très négatif à plutôt positif).** Cette question doit être sautée si le patient n'a pas de maladie chronique.

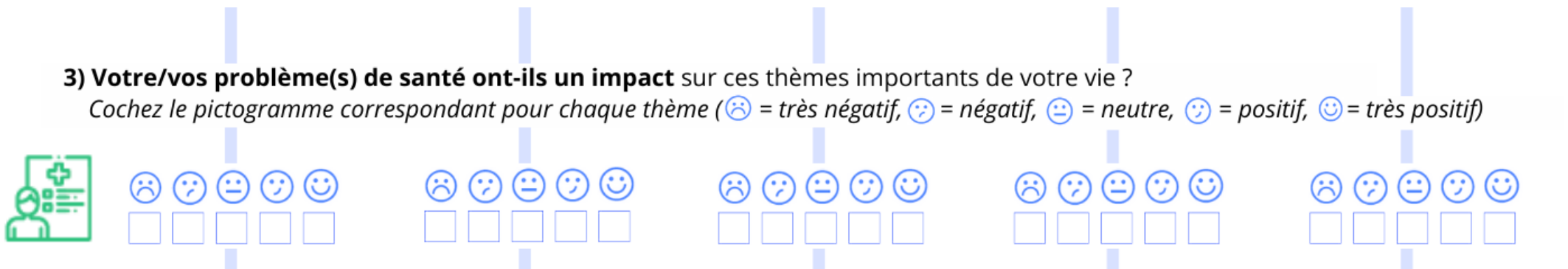

3) Votre/vos problème(s) de santé ont-ils un impact sur ces thèmes importants de votre vie ?
Cochez le pictogramme correspondant pour chaque thème (= très négatif, = négatif, = neutre, = positif, = très positif)

4. **Il indique enfin son ressenti de l'impact de ses traitements sur chacun des thèmes à l'aide 5 smileys (de plutôt négatif à très positif).** Cette question doit être sautée si le patient n'a pas de maladie chronique ou de traitement pour une maladie chronique.

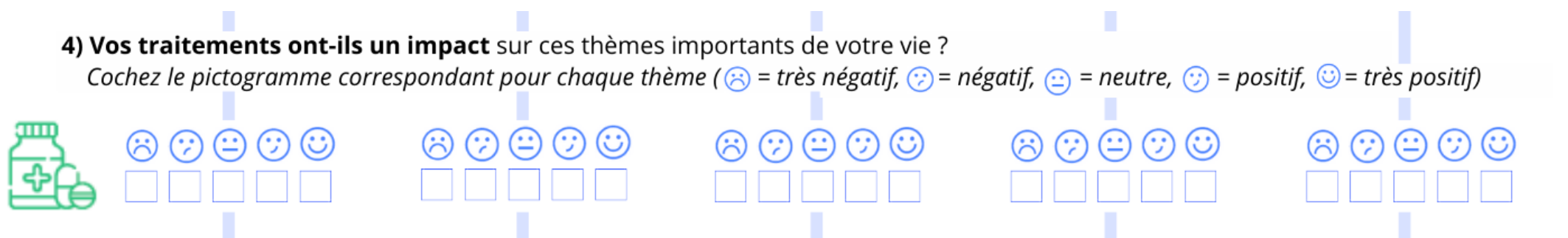

4) Vos traitements ont-ils un impact sur ces thèmes importants de votre vie ?
Cochez le pictogramme correspondant pour chaque thème (= très négatif, = négatif, = neutre, = positif, = très positif)

UTILISATION QUALITATIVE DU QUESTIONNAIRE

Le questionnaire PerQolateur a été conçu initialement pour une utilisation qualitative. Dans un tel cadre, l'idée était surtout pour le clinicien qui suit des patients de cibler les thèmes impactés **négativement** ( ) par la maladie et/ou par les traitements pour modifier la prise en charge thérapeutique. Cela permettait pour le clinicien de lancer un échange avec le patient pour mieux comprendre son ressenti négatif, sans se perdre dans une notion de qualité de vie très large, dans laquelle le clinicien pouvait se perdre ou ne pas percevoir son moyen d'agir.

UTILISATION QUANTITATIVE DU QUESTIONNAIRE

Une utilisation quantitative du questionnaire PerQolateur à l'aide de 3 scores :

- Le score de bien être
- Le score d'impact de la maladie
- Le score d'impact des traitements

LE SCORE DE BIEN ETRE

Il est calculé en calculant la moyenne des réponses à la question 2.

$$Score_{Bien-être} = \frac{\sum Q2}{n}$$

Avec n le nombre de thèmes remplis par le patient (5 normalement) et Q2 les réponses de bien-être (telles que remplies) aux n thèmes remplis. Le score est compris entre 0 et 10.

LE SCORE D'IMPACT DE LA MALADIE

Il est calculé en calculant la moyenne des réponses à la question 3.

$$Score_{Impact\_maladie} = \frac{\sum Q3}{n}$$

Avec n le nombre de thèmes remplis par le patient (5 normalement) et Q3 les réponses d'impact de la maladie aux n thèmes en utilisant les codes suivants : très négatif=-2, négatif=-1, neutre=0, plutôt positif=1 et très positif=2. Le score est compris entre -2 et 2. Les scores négatifs représentent des impacts négatifs de la maladie, et les scores positifs représentent des impacts positifs de la maladie.

LE SCORE D'IMPACT DES TRAITEMENTS

Il est calculé en calculant la moyenne des réponses à la question 4.

$$Score_{impact\_traitements} = \frac{\sum Q4}{n}$$

Avec n le nombre de thèmes remplis par le patient (5 normalement) et Q4 les réponses d'impact des traitements aux n thèmes en utilisant les codes suivants : très négatif=-2, négatif=-1, neutre=0, plutôt positif=1 et très positif=2. Le score est compris entre -2 et 2. Les scores négatifs représentent des impacts négatifs des traitements, et les scores positifs représentent des impacts positifs des traitements.

EXEMPLE

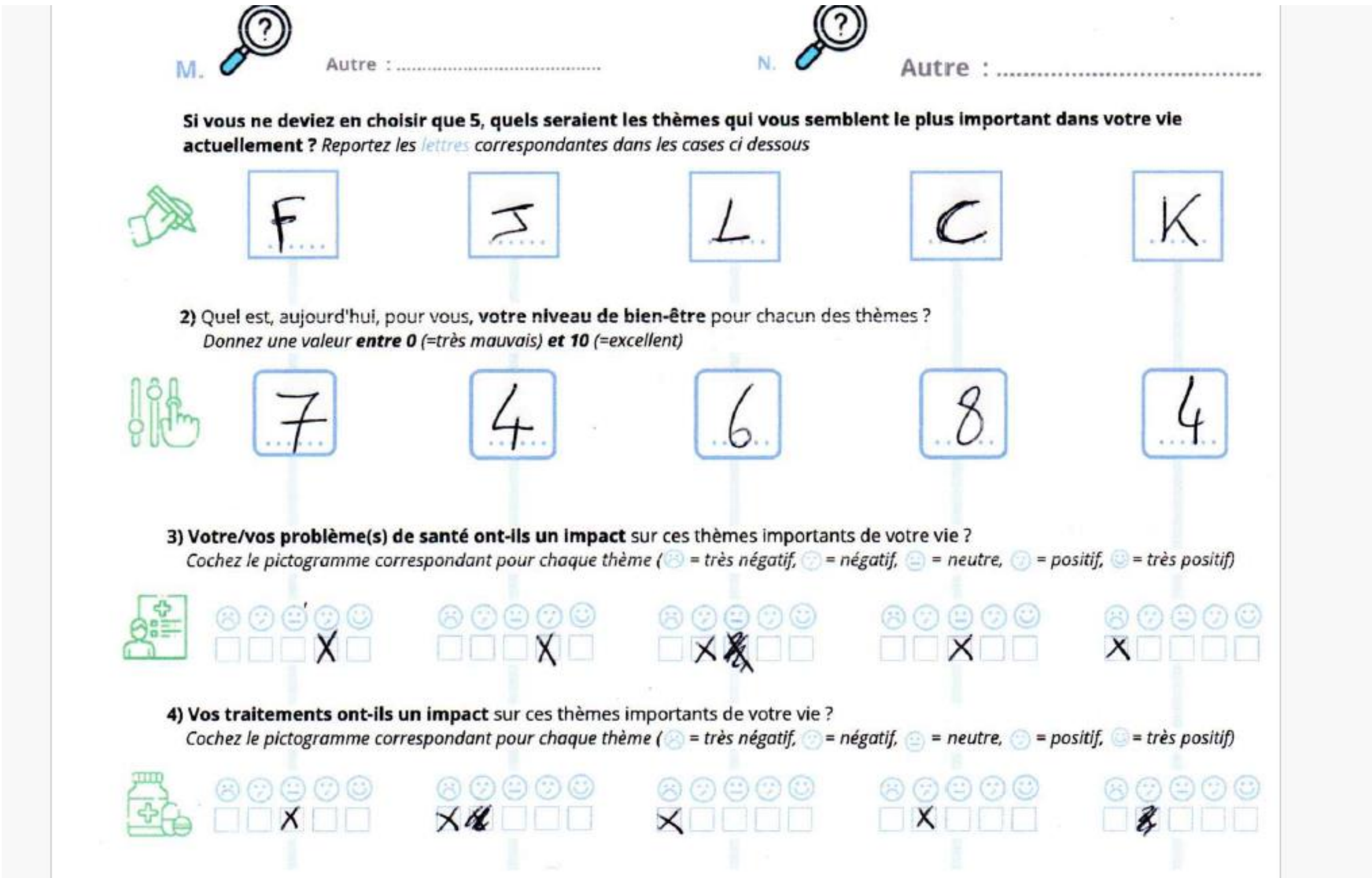

M. Autre : .... N. Autre : ....

Si vous ne deviez en choisir que 5, quels seraient les thèmes qui vous semblent le plus important dans votre vie actuellement ? *Reportez les lettres correspondantes dans les cases ci dessous*

F J L C K

2) Quel est, aujourd'hui, pour vous, **votre niveau de bien-être** pour chacun des thèmes ?
*Donnez une valeur **entre 0** (=très mauvais) **et 10** (=excellent)*

7 4 6 8 4

3) Votre/vos problème(s) de santé ont-ils un impact sur ces thèmes importants de votre vie ?
Cochez le pictogramme correspondant pour chaque thème (= très négatif, = négatif, = neutre, = positif, = très positif)

4) Vos traitements ont-ils un impact sur ces thèmes importants de votre vie ?
Cochez le pictogramme correspondant pour chaque thème (= très négatif, = négatif, = neutre, = positif, = très positif)

$$Score_{Bien-être} = \frac{7+4+6+8+4}{5} = \frac{29}{5} = 5.80$$

$$Score_{impact\_maladie} = \frac{1+1+(-1)+0+(-2)}{5} = \frac{-1}{5} = -0.20$$

$$Score_{impact\_traitements} = \frac{0+(-2)+(-2)+(-1)}{4} = \frac{-5}{4} = -1.25$$
